# Intracardiac left atrial tuberculoma in an eleven-month-old infant: case report

**DOI:** 10.1186/1471-2334-11-359

**Published:** 2011-12-30

**Authors:** Cantinotti M, De Gaudio M, de Martino M, Assanta N, Moschetti R, Veneruso G, Crocetti M, Murzi B, Chiappini E, Galli L

**Affiliations:** 1 G. Monasterio Tuscan Foundation, Heart Hospital, National Research Institute, Massa, Italy; 2 Department of Sciences for Woman and Child's Health, University of Florence, Anna Meyer Children's University Hospital, Florence, Italy

## Abstract

**Background:**

Cardiac tuberculosis is rare and usually manifests as tuberculous pericarditis. Involvement of other part of the heart is unusual and descriptions in the pediatric literature are confined to few case reports regarding mainly myocardial tuberculosis.

**Case presentation:**

We describe a case of pulmonary miliary tuberculosis associated with intracardiac left atrial tuberculoma in an immunocompetent eleven-month-old infant successfully treated with surgery and antituberculous therapy.

**Conclusion:**

Although unusual, involvement of endocardium in disseminated tuberculosis should be kept in mind.

## Background

Tuberculosis (TB) is one of the top ten causes of death among children worldwide and it is a direct consequence of adult TB [1]. In the natural history of childhood pulmonary TB, primary infection before two years of age frequently progresses to disease within twelve months [2]. Young age and human immunodeficiency virus type 1 (HIV-1) infection are the most important risk factors for severe or disseminated disease [2,3]. The involvement of the heart in TB is a very rare clinical condition both in adults and children [4,5]. We report here a case of pulmonary miliary TB associated with intracardiac left atrial tuberculoma in an eleven-month-old infant successfully treated with surgery and antituberculous therapy.

## Case presentation

An eleven-month-old female infant was referred to her local hospital for a 2-week history of intermittent fever, cough, dyspnea, night sweats and poor feeding. She was initially treated with amoxicillin-clavulanic acid for presumed upper respiratory tract infection without improvement.

**Figure 1:**
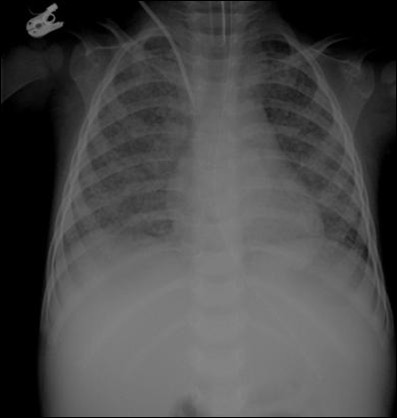
**Chest X-Ray**. Diffuse, bilateral, small lung nodules suggestive of acute miliary pulmonary tuberculosis.

On examination, she was underweight for age, had a temperature of 37.5°C, pulse was regular with a rate of 130 beats per minute, blood pressure was normal, transcutaneous oxygen saturation was 96% on room air, and respiration rate was 40 breaths per minute. A few crackles were heard bilaterally and a mild systolic murmur was noted. She had soft, mobile, non tender, small volume cervical lymph nodes with normal overlying skin and no evidence of discharge. The rest of the clinical examination was unremarkable. A complete blood count at presentation revealed a hemoglobin of 10.4 mg/dL, white cell count of 18 × 10^3 ^cell/μL with neutrophilia, and a platelet count of 660 × 10^3^cell/μL. Both biochemistry profile and C reactive protein levels were normal. HIV-1 serology was nonreactive. Chest X-Ray (CXR) revealed diffuse, bilateral, small lung nodules which could suggest acute pulmonary miliary TB (Figure 1). The tuberculin skin test (TST) and the QuantiFERON-TB Gold test were negative. Results of acid-fast bacilli (AFB) smears on three early morning gastric aspirates were negative. Polymerase chain reaction (PCR) for *Mycobacterium tuberculosis complex *and colture on gastric aspirates were pending. Family members were investigated for TB, but both parents had negative CXR and TST and the source of infection was not identified.

**Figure 2:**
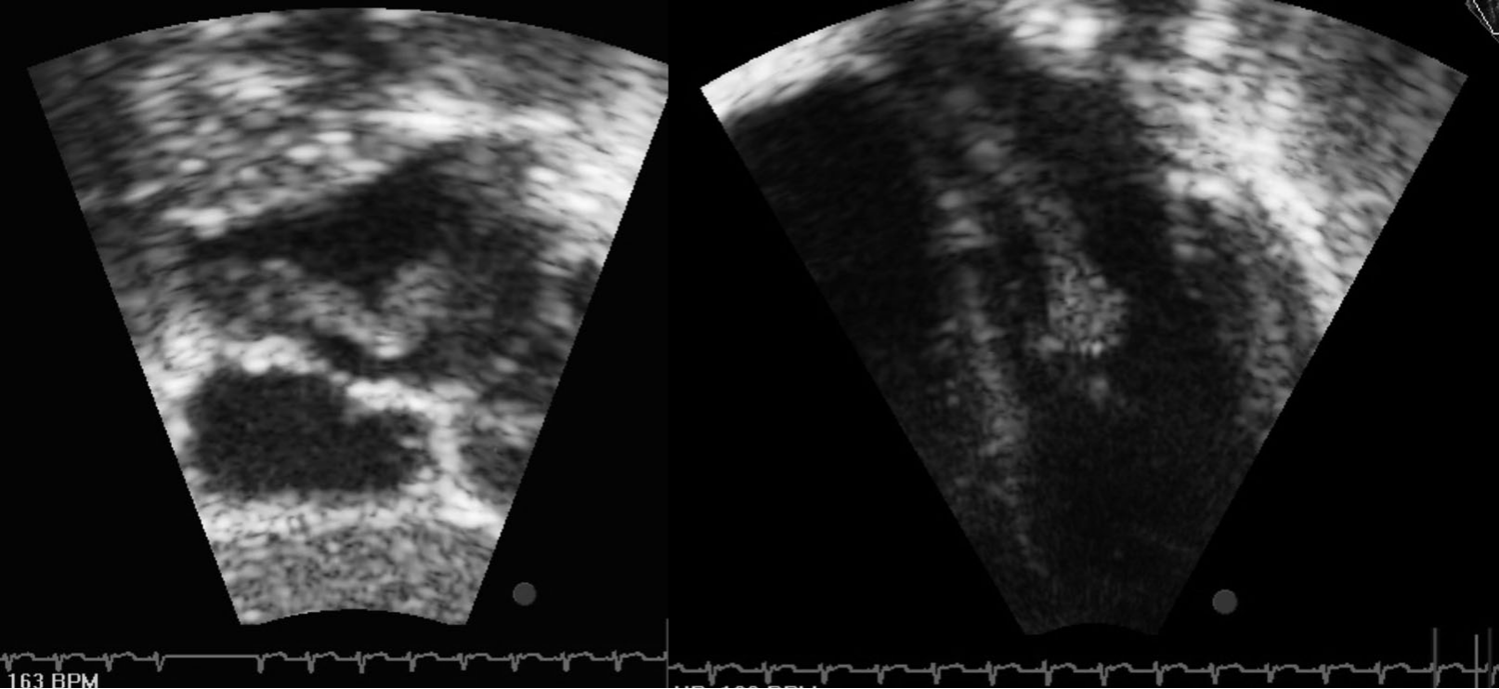
**Trans-thoracic echocardiographic subcostal and parasternal views**. A voluminous left atrial intracavitary pedunculated mass prolapsing during the diastole from the right lower pulmonary vein into the left ventricle through the mitral valve.

On day 3 after admission due to the presence of a mild systolic murmur and the persistence of intermittent dyspnea, echocardiography (ECHO) was performed and revealed a voluminous left atrial intracavitary pedunculated mass prolapsing during the diastole from the right lower pulmonary vein into the left ventricle through the mitral valve (Figure 2). No mitral stenosis and only trivial mitral regurgitation were noted. The patient was immediately transferred to the cardio-surgical unit and complete excision of an homogeneous and yellowish in color mass was performed through median sternotomy, under cardiopulmonary bypass. Histopathological examination of the removed mass revealed fibrotic tissue with mixed inflammatory cells and necrotic debris with a vaguely granulomatous appearance and AFBs were found. PCR for *Mycobacterium tuberculosis complex *was positive on the removed mass and on previous gastric aspirates.

**Figure 3:**
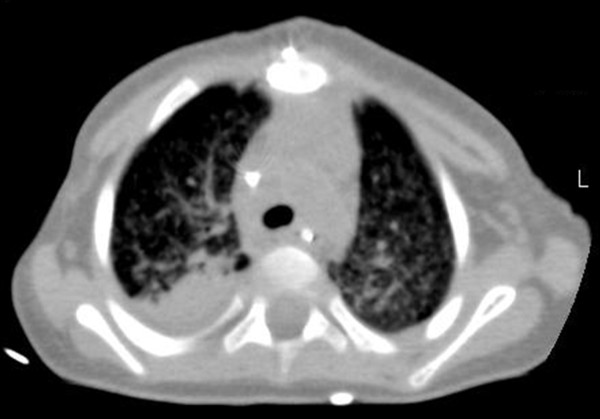
**Chest Computed Tomography**. Innumerable tiny, well-defined, miliary nodules throughout the lungs, and bronchovascular structures, a consolidation with air bronchogram in the posterior segment of the right upper lobe and an initial consolidation in the lateral segment of the right inferior lobe with evident local bronchiectasis.

Three days after surgery, a chest computed tomography (CT) scan revealed widespread miliary nodules, compatible with acute pulmonary miliary TB and showed a right upper lobe consolidation (Figure 3). The cranial contrast-enhanced CT scan and the abdominal ultrasound were normal.

Antituberculous therapy with streptomycin (20 mg/kg intramuscularly once daily), isoniazid (10 mg/kg orally once daily), rifampicin (15 mg/kg orally once daily), ethambutol (20 mg/kg orally once daily), pyrazinamide (30 mg/kg orally once daily) and corticosteroids were started immediately with remarkable improvement in her symptoms in two weeks. Streptomycin was suspended once the drug susceptibility results showed no resistance to the first-line antituberculous drugs. Coltures of gastric aspirates and tissue specimens of the removed mass confirmed *Mycobacterium tuberculosis*. Further investigations on possible source case found a Romanian grandmother with active pulmonary TB.

Echocardiograms were performed at regular postoperative intervals and no residual lesions were noted. The infant completed 1 year of antituberculous therapy, recovered completely, and did well during the whole follow-up.

## Discussion

To our knowledge, this is the youngest patient reported with a cardiac tuberculoma and the first infant with an endocardial tuberculoma in a left heart chamber successfully treated.

Cardiac TB is a rare disease and it most usually manifests as tuberculous pericarditis [3]. Involvement of other parts of the heart is unusual and descriptions in the pediatric literature are confined to few case reports regarding mainly myocardial TB [5-8]. Myocardial TB may occur secondary to haematogenous spread from a remote tuberculous focus, lymphatic spread from mediastinal lymph nodes, or direct involvement from the adjacent pericardium [5]. Three distinct forms of myocardial TB have been recognized: miliary, diffuse infiltrating, or nodular with central caseation or tuberculoma [5,9]. Tuberculoma, a mass-like manifestation of the disease, was first reported by Morgagni in 1761 [7,9,10]. Myocardial tuberculomas are mostly located in the right heart and particularly in the wall of the right atrium [4]. They are usually sharply demarcated from the surrounding parenchyma and may be single or multiple [4,9].

The infant we described had a miliary pattern pulmonary TB. The infection may have spread hematogenously to the left heart chambers causing a single voluminous left atrial endocardial tuberculoma. Tuberculoma of the heart has been associated to congenital heart diseases in children, but our patient had no anatomic abnormalities [6]. In addition, disseminated TB is often associated with acquired immunodeficiency syndrome, but our patient was immunocompetent and negative for HIV-1 [1].

Clinically, cardiac tuberculomas may be asymptomatic, or presenting with pulmonary vein obstruction due to left atrial mass lesions, left ventricular aneurysm, right ventricular outflow tract obstruction, superior vena cava obstruction, coronary artery occlusion, impairment of ventricular contractility, ventricular rupture, aortic insufficiency or regurgitation, cardiac arrhythmia, complete heart block, and sudden cardiac death [8,9,11,12]. Our patient presented only a systolic murmur and intermittent dyspnea.

The treatment of cardiac TB is, first of all, antituberculous therapy [7]. Complete recovery of patient's signs and symptoms along with imaging evidence of cardiac involvement resolution on antituberculous therapy has been reported previously [8]. However, if the diagnosis remains questionable or in cases of severe hemodynamic compromise, refractory arrhythmias or threatening thromboembolism, the surgical resection of tuberculoma may be considered [9,13]. In 1997, Shoeman JF et al. described an infant with miliary TB and an acute stroke caused by an infected thromboembolus which arose from a suspected endocardial tuberculous vegetation [14]. In our patient the voluminous tuberculoma prolapsing during the diastole had a high risk of systemic embolization and a surgical resection was immediately performed. Subsequently, a 12-month course of antituberculous therapy to prevent hematogenous dissemination and to treat pulmonary parenchymal disease was completed with total recovery.

The infant we described had a CXR suggestive of acute miliary pulmonary TB which was supported by the chest CT performed few days later. However TST, QuantiFERON-TB Gold test, and microscopic examination of gastric aspirates were negative. Only PCR and coltures of gastric aspirates and tissue specimens of the removed mass revealed *Mycobacterium tuberculosis*. This confirms that up to 40% of immunocompetent young children with colture-documented active TB do not react to the TST, nor have a positive QuantiFERON-TB Gold test, and have a less than 15% probability of having an AFB smear positive gastric aspirate [1,15-17].

## Conclusions

The peculiarity of TB in the pediatric population is the imperceptible and often rapid progression from infection with *Mycobacterium tuberculosis *to active disease [1]. As a matter of fact, the risk of progression depends on various factors, including age at exposure, nutritional and immune status, genetic factors, virulence of the organism, and magnitude of initial infection [1]. Pulmonary parenchymal disease and intrathoracic adenopathy are the most common clinical manifestations of pediatric TB, accounting up to 80% of all cases [2,16]. Among extrapulmonary manifestations, cardiac TB is extremely rare. However, negative QuantiFERON-TB Gold test, TST, and microscopic examination on gastric aspirates should not dissuade pediatricians from further investigations in infants for active TB. Although rare, disseminated TB with heart involvement should be suspected in immunocompetent infants with an intracardiac mass.

## Competing interests

The authors declare that they have no competing interests.

## Authors' contributions

CM and DGM drafted the manuscript together and should be both considered first author. dMM, EC and VG participated in the design of the paper and revised it critically for the infectious diseases content. BM, AN, MR and MC participated in the design of the paper and revised it critically for the cardio-surgical content. LG help to draft the manuscript and revised the final version of the manuscript. All authors read and approved the final manuscript.

## Consent

Written informed consent was obtained from the parents for publication of this case report and any accompanying images. A copy of the written consent is available for review by the Editor-in-Chief of this Journal.

## Pre-publication history

The pre-publication history for this paper can be accessed here:

http://www.biomedcentral.com/1471-2334/11/359/prepub
